# Temporal Patterns of Wearable Accelerometer-Measured Physical Activity and Symptom Worsening in Knee Osteoarthritis: A 2-Year Longitudinal Study from the Osteoarthritis Initiative

**DOI:** 10.3390/s26030982

**Published:** 2026-02-03

**Authors:** Junichi Kushioka, Ruopeng Sun, Matthew Smuck

**Affiliations:** 1 Spine and Scoliosis Center, Shonan Fujisawa Tokushukai Hospital, Fujisawa 251-0041, Japan; junichi.kushioka@gmail.com; 2 Department of Orthopaedic Surgery, Osaka University, Osaka 565-0871, Japan; 3 Department of Orthopaedic Surgery, Stanford University, Stanford, CA 94063, USA; msmuck@stanford.edu; 4 Division of Physical Medicine and Rehabilitation, Stanford University, Stanford, CA 94063, USA

**Keywords:** knee osteoarthritis, physical activity, accelerometer

## Abstract

This study investigates the link between changes in physical activity (PA) measured by wearable accelerometers and the worsening of knee osteoarthritis (KOA) symptoms over two years. Using data from 782 participants in the Osteoarthritis Initiative accelerometer sub-study, PA was tracked with hip-worn ActiGraphs. Participants were classified as “worsening” if their Western Ontario and McMaster Universities Osteoarthritis Index (WOMAC) total score increased by >10 points and as “stable” otherwise. PA was categorized into daily counts and minutes spent in various intensity levels, and analyzed in 3 h intervals across the day. Of the participants, 123 (15.7%) experienced worsening symptoms. At baseline, both groups had similar characteristics aside from slower sit-to-stand times in the worsening group. Over two years, the worsening group had a greater decline in total daily activity counts (−18% vs. −10%) and more significant reductions during late afternoon and evening (15:00–21:00; −21% vs. −6%). This group also showed a notable decrease in gait speed, longer sit-to-stand times, and a trend towards greater medial joint space narrowing. These findings suggest that larger declines in PA, especially in activities in the late afternoon and evening, are associated with worsening KOA symptoms, although causality cannot be established.

## 1. Introduction

Knee osteoarthritis (KOA) is a highly prevalent degenerative joint disease in middle-aged and older adults and is a leading cause of pain, mobility limitation, and disability worldwide [1,2]. Pain, stiffness, and functional limitations substantially impair quality of life and complicate rehabilitation in affected patients [3]. Clinical guidelines consistently recommend low-impact aerobic exercise and physical activity (PA) as core components of KOA management [4]. However, the optimal “dose” and pattern of PA for patients with established KOA remain uncertain [5]. While some studies suggest that higher PA levels can improve symptoms and function [6], mechanical loading during certain activities may accelerate structural deterioration of the knee joint in susceptible individuals [7,8]. Clarifying how PA relates to both symptom trajectories and joint changes is therefore a clinically important question for physicians managing KOA.

Traditionally, PA in KOA has been evaluated using self-reported questionnaires or simple summary metrics from wearable devices, such as average daily step count or time spent in predefined intensity categories [9]. More recently, wearable accelerometers have enabled continuous, objective monitoring of PA and its temporal accumulation across the day. In geriatric and epidemiological research, such sensor-derived temporal patterns have been linked to frailty, functional decline, and mortality risk [10]. However, it remains unclear whether and how diurnal PA patterns capture clinically meaningful aspects of disease course in musculoskeletal conditions such as KOA. From a clinical perspective, understanding these patterns could help explain why some patients reduce activity preferentially at specific times of day (for example, when pain or fatigue peaks), and may inform more individualized PA prescriptions and rehabilitation strategies.

This study utilizes data from the Osteoarthritis Initiative (OAI) accelerometer sub-study [11] to examine the association between sensor-measured PA and 2-year worsening of KOA. Specifically, we compare changes in overall PA levels and diurnal PA accumulation patterns between patients with stable versus worsening symptoms, as defined by the Western Ontario and McMaster Universities Osteoarthritis Index (WOMAC). We also explore parallel changes in functional performance measures and radiographic joint space narrowing. Our aim is to generate clinically interpretable evidence, based on wearable sensor data, that can support physicians in tailoring PA recommendations and in designing future clinical trials targeting activity-based interventions for KOA.

## 2. Materials and Methods

### 2.1. Study Design and Participants

This study is based on a secondary analysis of data from the OAI accelerometer sub-study, a multicenter, longitudinal, prospective observational cohort of adults with or at high risk for symptomatic KOA, available at https://nda.nih.gov/oai/ (accessed on 5 January 2023) [11]. Between 2004 and 2006, 4796 participants aged 45–79 years were recruited at four clinical sites in the United States, with institutional review board approvals and written informed consent obtained at each site.

The OAI protocol details the recruitment process and the inclusion/exclusion criteria for three primary cohorts. Briefly, participants were assigned to one of three cohorts: (1) a Progression Cohort with radiographically confirmed KOA and frequent knee symptoms; (2) an Incidence Cohort at increased risk for developing KOA; and (3) a healthy Reference Cohort without major risk factors or clinical evidence of KOA.

**Figure 1:**
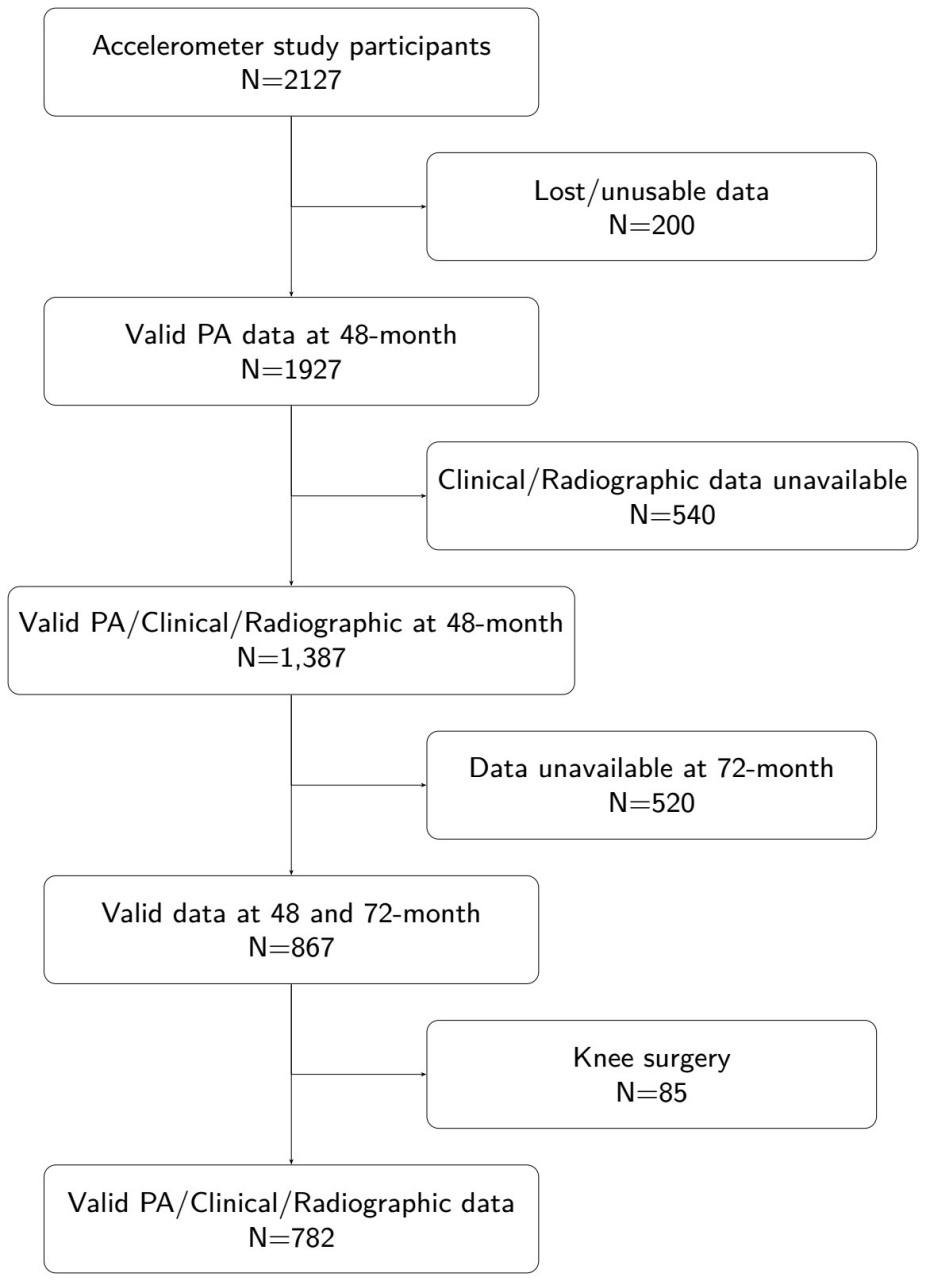
Flow chart of patient data extraction from OAI dataset.

Of the 4796 OAI participants, 2127 were invited to participate in an accelerometer sub-study at the 48-month visit. Details of the accelerometer protocol and sample characteristics have been reported previously [12]. Notable research findings from the OAI accelerometer data indicate that despite substantial health benefits from physical activity, adults with knee OA were particularly inactive based on objective accelerometry monitoring [12], as well as the finding that moderate-to-vigorous physical activity (MVPA) does not increase the risk of joint worsening [13]. Additionally, robust activity patterns in the morning or evening are linked to a lower risk of functional decline [14]. For the present analysis, we included participants who had valid clinical, functional, accelerometry, and radiographic data at both the 48-month (baseline for this analysis) and 72-month (2-year follow-up) visits. We excluded individuals who had undergone knee replacement surgery before the 48-month visit (details of the characteristics of individuals who are excluded from the analysis due to having knee surgery are presented in a Supplementary Table S1), lacked radiographic readings, or had missing covariates at baseline. After these exclusions, 782 participants with complete data at both time points (and without knee replacement at the timing of OAI enrollment) remained for analysis (Figure 1).

### 2.2. Clinical and Radiographic Outcome Measurements

Demographic data (age, gender, race, body mass index [BMI]) and clinical outcomes were collected at the 48-month and 72-month visits. Patient-reported outcomes included the WOMAC, the Center for Epidemiologic Studies Depression Scale (CESD), the Physical Activity Scale for the Elderly (PASE), and the number of falls over the preceding 12 months. Objective physical function measures included usual gait speed (20-m walk test) and the five-times sit-to-stand test (5TST, seconds).

Radiographic outcomes focused on medial minimum joint space width (JSW [mm]) and the Kellgren–Lawrence (KL) grade, with emphasis on the medial compartment due to its higher susceptibility to KOA [15]. Medial minimum JSW was chosen for its relevance in reflecting KOA’s structural pathology [16]. When JSW data were missing at a given time point, we reviewed all available radiographs across the ten OAI visits. For absent JSW measurements, we imputed the missing time point by calculating the mean value from adjacent visits, provided that at least one adjacent measurement was available. KL grades were obtained from centrally read radiographic assessments. For participants with bilateral KOA, we defined the “worse side” as the knee with the higher KL grade; if KL grades were equal, the knee with the smaller JSW was considered worse. Missing KL grades were inferred from the previous visit, under the assumption of condition stability. All subsequent analyses used the radiographic and clinical characteristics of this worse knee, as it is more likely to drive symptoms and predominantly influences functional and activity outcomes.

### 2.3. Measurement of Physical Activity

Physical activity (PA) was measured using the ActiGraph GT1M monitor (ActiGraph LLC, Pensacola, FL, USA). The device was worn on an elastic belt over the right hip and recorded vertical accelerations as activity counts per minute, reflecting the intensity of bodily movements in the vertical plane.

Participants were instructed to wear the accelerometer for seven consecutive days during waking hours, removing it only for water-based activities such as bathing or swimming. Non-wear time was defined as intervals of ≥90 consecutive minutes with zero counts, allowing for up to 2 min of counts below 100 counts/min within this interval, as per OAI protocols [17]. Days with at least 10 h of wear time were considered valid, and participants with 4–7 valid days were included in the analysis [18]. It is worth noting that we did not separate weekday and weekend in this analysis to maximize our sample size, as not all individuals have valid weekend data during the 7-day recording period.

PA outcomes included in this analysis were the following: total daily step counts, total daily activity counts, and time spent in three activity intensity categories, defined using conventional cut-points for older adults (Freedson’s intervals [19]: (1) moderate-to-vigorous physical activity (MVPA, >1952 counts/min), (2) light physical activity (LPA, 100–1951 counts/min), and (3) sedentary time (SED, 0–99 counts/min)).

To characterize diurnal variations in PA, we segmented the 24 h day into eight consecutive 3 h intervals (00:00–03:00, 03:00–06:00, …, 21:00–24:00). For each interval, we calculated activity counts, step counts, and time spent in MVPA, LPA, and SED. We also calculated the percentage of non-wear time within each interval (Supplementary Figure S1). Non-wear time was minimal between 09:00 and 21:00, and our primary analyses therefore focused on four consecutive daytime intervals—09:00–12:00 (morning), 12:00–15:00 (early afternoon), 15:00–18:00 (late afternoon), and 18:00–21:00 (evening)—which we considered most representative for examining daily PA patterns.There are individuals who are also active before 9:00 or 21:00, however, due to large percentage of non-wearing data; to ensure limited bias of activity estimate, we decided to include only 09:00–21:00.

### 2.4. Grouping Based on KOA Progression

Participants were categorized into two groups according to changes in KOA-related symptoms over the 2-year period. The “Worsening” group comprised individuals who showed an increase of >10 points in their WOMAC total score between 48 and 72 months, indicating a deterioration exceeding the minimal clinically important difference (MCID) for KOA [20]. The “Stable” group included participants whose WOMAC total scores changed by less than 10 points over the same interval.

### 2.5. Statistical Analysis

We compared baseline characteristics and 2-year changes between the “Stable” and “Worsening” groups. Data normality was assessed using the Shapiro–Wilk test. Between-group comparisons were performed using independent t-test for normally distributed variables and the Mann–Whitney U test for non-normally distributed variables. Categorical variables were compared using chi-square tests. For time-of-day analyses of PA, we examined absolute values at baseline and at 2-year follow-up, as well as within-person change scores for each 3 h interval, and compared these changes between groups. We considered a two-sided *p*-value < 0.05 to indicate statistical significance. All data processing and statistical analyses were conducted in Python 3.9 (Python Software Foundation) using standard scientific libraries (pandas, NumPy, SciPy, and matplotlib).

## 3. Results

### 3.1. Baseline Characteristics

Table 1 outlines the baseline characteristics of the 782 participants. The majority (85.8%) self-identified as White or Caucasian, and 55.5% were female. On average, participants showed moderate disability related to KOA (average WOMAC total score: 11.9). Functional tests indicated a normal average gait speed (1.34 m/s, above the 1.2 m/s threshold established from prior research based on older adults [21,22] and adults with knee OA [23]) and a moderate ability in the five-times sit-to-stand test (average: 10.66 s, also on par with reference values for the corresponding age group [24], and well below the established cutoff of 15 s, that has been linked to increased fall risk [25]). Radiographic data showed that 75.6% had radiographic KOA (KL grades ≥ 2). Regarding physical activity, light activity was more common (average LPA: 278.1 min/day), while MVPA was less frequent (average MVPA: 19.7 min/day). In the present study, baseline activity metrics including daily total minutes in SED, LPA, and MVPA are on par with numbers reported in prior studies [12,26,27]; see Supplementary Table S2 for details.

### 3.2. Comparison of Stable vs. Worsening Groups

In our study of 782 participants, 659 (84.3%) were classified as “Stable”, while 123 (15.7%) fell into the “Worsening” group. Initially, both groups displayed similar baseline characteristics, particularly in terms of age and BMI, as shown in Table 2.

At the baseline, there is no significant difference between two groups in any demographics, clinical outcomes, radiographic outcomes, or physical activity outcomes, except the five-times sit-to-stand performance, with the the Stable group averaging 10.50 s and the Worsening group averaging 11.53 s (*p* = 0.03). The majority in both groups had KL grades ≥2, and no significant differences were observed in WOMAC scores or gait speed. At follow-up, compared with the Stable group, the Worsening group exhibited greater deterioration in gait speed and five-times sit-to-stand performance (*p* < 0.01). Self-reported physical activity (PASE) and depressive symptoms (CESD) remained similar between the two groups.

In terms of accelerometer-measured PA at baseline, both groups had similar levels of daily total activity counts and minutes of activity within the different intensity intervals. During the follow-up, the Worsening group showed marginal lower daily total activity counts (*p* = 0.07) and significantly fewer minutes of MVPA (*p* = 0.02), with no significant differences in SED or LPA levels. Longitudinally, both groups reported reductions in daily total activity counts over time, with the Worsening group experiencing a more pronounced decline (Worsening 18%, Stable 10%, *p* = 0.02). The decrease in MVPA was also significantly larger in the Worsening group (Worsening 28%, Stable 13%, *p* = 0.049), while interval changes in SED and LPA were not significant. Among all physical activity intensities, MVPA showed the most pronounced difference between the groups.

### 3.3. Comparison of Stable vs. Worsening Groups: Temporal Pattern of Physical Activity

**Figure 2:**
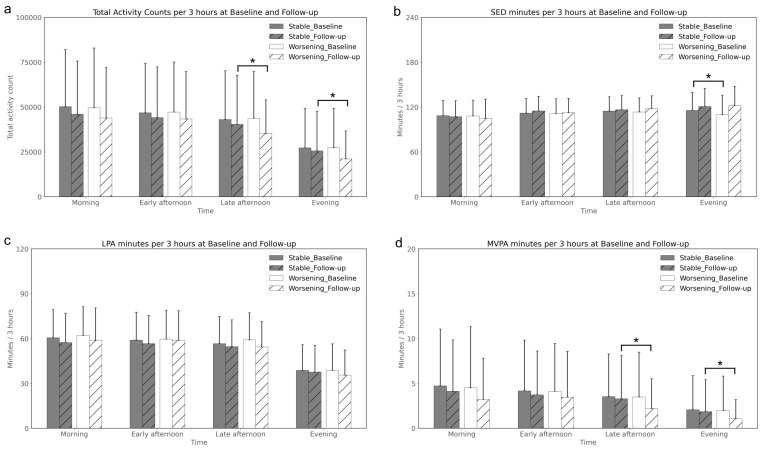
Temporal analysis of physical activity per 3 h at baseline and follow-up. (**a**) Total Activity Counts, (**b**) SED minutes, (**c**) LPA minutes, (**d**) MVPA minutes. The * identifies a significant difference between the Stable and the Worsening groups (*p* < 0.05). (SED; Sedentary, LPA; Light Physical Activity, MVPA; Moderate-to-Vigorous Physical Activity).

A general decline in total activity count across all 3 h intervals from baseline to follow-up was observed for both groups, as illustrated in Figure 2a. The only significant interval reductions were observed in the Worsening group for activity during late afternoon and evening, as further detailed in Supplementary Figure S2.

Sedentary time patterns, shown in Figure 2b, demonstrate a non-significant and small interval decrease in sedentary time during the morning and a small increase from early afternoon onwards in both groups. At baseline, the Worsening group had significantly less sedentary time during the evening period compared to the Stable group.

Figure 2c, focusing on LPA, revealed a uniform decrease in LPA at follow-up for both groups. There were no significant differences between the groups in LPA at any time point or over the time interval.

The trends in MVPA, as depicted in Figure 2d, mirrored the total activity count patterns, with both groups demonstrating a consistent decrease in MVPA over time. The Worsening group experienced a more notable reduction at follow-up, particularly during late afternoon and evening periods where the change was statistically significant.

Overall, while both groups experienced a decline in physical activities over time, the Worsening group demonstrated a more substantial reduction in MVPA, particularly during the late afternoon and evening periods.

## 4. Discussion

This study investigated the association between physical activity and changes in KOA progression over a 2-year period, focusing on how changes in PA levels and the temporal pattern of PA accumulation differed between clinically stable and worsening patients with KOA. In this study, the clinically Worsening group was defined by participants reporting an interval increase in their WOMAC total scores that surpassed the MCID [20]. Our classification method led to 15.7% of participants qualifying for placement in the Worsening group, while the remaining 84.3% comprised the Stable group. Interestingly, these findings are in close agreement with those of a previous study that monitored the progression of KOA over eight years [28]. In that study, 14% of participants were categorized as experiencing worsening WOMAC pain scores through a mixed-effects mixture model approach. This parallelism suggests a consistent pattern in the self-reported progression of KOA, supporting the robustness of these observed trends despite the differences in analytical techniques and the observational nature of both studies.

Over the 2-year period, the Worsening group demonstrated larger declines in total activity counts (−18.1% vs. −10.0 %) and functional capacity (walking speed, −5.0% vs. −1.1%; sit-to-stand, +4.9% vs. +0.3%) compared to the Stable group, even though no differences were observed at baseline evaluation between the 2 groups among all measures, except the sit-to-stand test outcome (Worsening group was slower). This observation aligns with the known trajectory of KOA, where worsening symptoms often are associated with reduced physical function and reduced physical activity [29,30]. As physical function deteriorates, a corresponding decrease in physical activity is observed [12], although it is also plausible that reduced activity further contributes to functional decline; the present observational analysis cannot determine the direction of these associations. Previous research using the OAI dataset showed that higher pain severity was related to worse chair stand and 400 m walk test performance [31]. Further study [29] using the OAI dataset confirmed that the presence of pain can independently and negatively influence physical function test performance, and that symptoms are more important than structural characterization of OA. The study [29] also revealed that the addition of patellofemoral KOA to a tibiofemoral KOA was not a significant factor in the models, again suggesting that symptoms are more important than the structural changes of OA in determining physical function.

Our detailed analysis of physical activity intensity highlighted greater decrease over time in MVPA as an important characteristic for the Worsening KOA group, whereas SED and LPA minutes were comparable between the two groups. At baseline, MVPA levels were comparable between the groups, but at 2-year follow-up the Worsening group revealed a more significant decrease over time in MVPA. This suggests that decreases over time in MVPA are associated with self-reported worsening KOA symptoms. Although the proportion of MVPA in a day is very small, the amount of time spent in MVPA appears to be an important marker of KOA symptom progression. Whether it is a cause or a consequence of the worsening symptoms remains to be determined. Thus, future studies on KOA symptom management that explicitly examine the effects of modifying time spent in MVPA are needed to explore the causal nature of this relationship, and potentially to help in the development of disease-specific PA guidelines in KOA. A previous study similarly highlighted the important link between MVPA and KOA (using a Random Forest classifier and Shapley values for feature interpretation on a two-year dataset), finding that lower minutes spent in MVPA were the most significant predictor for gait decline [32]. This finding aligns with the observation that reductions in more vigorous activities are more closely linked with deterioration in functional mobility, particularly gait, in KOA patients. In contrast, sedentary time and low-intensity physical activity were not identified as key predictors of gait status. Yet another study supported this, reporting no significant differences in sedentary time between KOA patient groups with normal or improved gait function [33].

Unique to our study was the evaluation of activity accumulation during the day. We found that the reduction of physical activity in the Worsening group is centered between the late afternoon and evening periods, as shown in the total activity count and the MVPA analysis. Higher afternoon activity has been previously linked to reduced all-cause mortality [34,35], potentially due to the link to metabolic benefits of higher body temperature [36], and better glucose disposal [37] in the afternoon. The reduced afternoon activity, as shown in this study, could be attributed to overall physical function/health decline that is corresponding to the worsening symptoms from self-reported WOMAC. A longitudinal study of aging showed a 1.3% decrease in overall physical activity counts annually from mid-to-late life [38]. It was observed that morning activity levels remained relatively stable across age groups, whereas older individuals demonstrated notably lower activity levels in the afternoon and evening [38]. Another study [39] focusing on middle-aged women also found a decline in activity as the day progressed, suggesting that a low threshold for fatigue may contribute to a sedentary lifestyle and its associated age-related consequences. We believe our study is the first to document this specific pattern of lower physical activity in the later parts of the day among individuals with knee OA. This unique observation may be related to increased pain and a lower threshold for fatigue due to the worsening symptoms and poor functional performance of knee OA. However, we acknowledge that the time-of-day signal should not be interpreted as a causal factor or as implying that afternoon/evening activity is uniquely critical to knee OA progression. Instead, it is essential to build upon the evidence supporting the broader principle of maintaining consistent physical activity throughout the day for knee OA management/care [40,41,42,43,44]. We believe this interesting observation merits further investigation with prospective study designs to explore the underlying mechanisms of these patterns. Continued research in this area can enhance our understanding and improve management strategies for knee OA.

In the context of KOA management, recent guidelines by EULAR, ACR, and OARSI underscored the importance of physical activity, particularly structured exercise programs that focus on muscle strengthening, joint mobility, proprioception, and aerobic exercises, as a cornerstone in the management of osteoarthritis [30]. To enhance the effectiveness of these interventions, it is essential to adapt both the intensity and timing of physical activities according to individual needs. This study’s findings, revealing specific patterns in physical activity among individuals with KOA, offer descriptive insight for future intervention strategies. Understanding the effects of different activity intensities and times of day on individuals with KOA may contribute to the development of more effective, tailored physical activity guidelines. The duration of MVPA is likely to be relevant for maintaining physical function and managing KOA symptoms, but our observational data do not allow firm conclusions about optimal thresholds. Therefore, increasing the duration of MVPA should be considered a hypothesis that requires testing in appropriately designed interventional studies, rather than a direct recommendation for disease management. Additionally, our results have shown that physical activity levels change in KOA patients during late afternoon and evening periods (and mimic changes observed in the elderly). This suggests that interventions to increase physical activity might be more effective when timed accordingly, but this also requires prospective evaluation. Future study is needed to evaluate whether these tailored strategies enhance KOA management and lead to improved health outcomes and quality of life for the patients.

This study has several limitations. Firstly, the two-year interval was insufficient to observe large anatomic changes in the knee joint, particularly in terms of radiographic changes. This limited timeframe restricted our ability to thoroughly assess the long-term impact of physical activity on the structural progression of KOA. Therefore, future studies should consider a longer observation period to provide a more detailed understanding of how physical activity relates to knee joint deterioration over time. Secondly, we also acknowledge that weekday and weekend might have different patterns of physical activity; however, as not every individual included in the study has valid weekend data, we decided to pool both weekday and weekend together for this analysis. Future studies with multi-week data collection might be able to separate weekday and weekend data for more detailed physical activity profiling. We also recognize that in this work, the stable and worsening group was not exactly matched to each other based on demographics and baseline KL grade. However, as baseline demographic and KL score are not significantly different between the stable and worsening group, we believe that including all available participants in the stable control group would maximize statistical power and ensure that our findings are generalizable to a broader population. Fourthly, the challenge of accurately measuring the small space within the knee joint presents a risk of measurement errors. These potential inaccuracies could impact the findings of the study. Additionally, this study is an observational cohort design, which contrasts with an interventional approach. This design limits our ability to draw direct causal inferences about the associations we observed. In addition, our analytic sample combined participants from the OAI Progression, Incidence, and Reference cohorts, introducing heterogeneity in baseline KOA status and risk. We used relatively simple group-based comparisons rather than more advanced multivariable or mixed-effects models, and residual confounding by baseline symptoms, function, or comorbidities cannot be excluded. To address these issues, future research should focus on intervention studies and more comprehensive longitudinal modeling that meticulously examine how changes in time spent in different PA intensity intervals and the timing of physical activity affect KOA symptoms and structural outcomes. These studies would provide more definitive insights into the effectiveness of specific physical activity recommendations in managing KOA.

## 5. Conclusions

This study has provided descriptive insights into the relationship between physical activity patterns and the progression of KOA symptoms. We observed a significant decrease over time in physical activity, particularly in MVPA, during late afternoon and evening among individuals with worsening KOA. This unique observation may be related to increased pain and a lower threshold for fatigue due to the worsening symptoms and poor functional performance of knee OA. However, we acknowledge that the time-of-day signal should not be interpreted as a causal factor or as implying that afternoon/evening activity is uniquely critical to knee OA progression. As such, our results should be regarded as hypothesis-generating, and the intensity, duration, and timing of PA interventions need to be evaluated in future studies before specific treatment strategies can be recommended.

## Figures and Tables

**Table 1:** Baseline Characteristics.

Demographic			Clinical		
Number		782	WOMAC total		11.9 (14.9)
Age		64.9 (9.1)	CESD		6.3 (7.2)
Gender	Female, n (%)	434 (55.5)	PASE		159.6 (80.9)
Race	White or Caucasian, n (%)	671 (85.8)	Cormorbidity ≥ 2	n (%)	86 (11.0)
	Black or African American, n (%)	102 (13.0)	Fall History ≥ 2	n (%)	117 (15.0)
	Asian, n (%)	4 (0.5)	Gait speed	[m/s]	1.34 (0.20)
	Other Non-white, n (%)	5 (0.6)	Five times sit to stand	[s]	10.66 (4.14)
**Physical Activity**			**Radiographic**		
Daily total activity counts	[$10^{4}$ count/day]	22.1 (10.7)	Minimum medial JSW	[mm]	3.78 (1.45)
SED	[min/day]	579.8 (81.8)	KL grades ≥ 2	n (%)	591 (75.6)
LPA	[min/day]	278.1 (75.8)			
MVPA	[min/day]	19.7 (19.5)			

The values represent the mean and (SD) for the continuous variables or n (%) for the categorical variables. WOMAC—Western Ontario and McMaster Universities Osteoarthritis Index; CESD—the Center for Epidemiologic Studies Depression Scale; PASE—the Physical Activity Scale for the Elderly; SED—Sedentary time; LPA—Light Physical Activity; MVPA—Moderate-to-Vigorous Physical Activity; JSW—medial-side minimum joint space width.

**Table 2:** Outcomes at baseline and follow-up, and longitudinal changes.

		Baseline			Follow-Up			Longitudinal Change		
		Stable	Worsening	*p*	Stable	Worsening	*p*	Stable	Worsening	*p*
**Demographics**										
Number	n (%)	659 (84.3)	123 (15.7)	NA						
Age		64.8 (8.9)	65.9 (9.8)	0.26						
Gender	Female, n (%)	364 (55.2)	70 (56.9)	0.81						
Race	White, n (%)	561 (85.1)	110 (89.4)	0.09						
**Clinical Outcomes**										
WOMAC total		12.1 (15.5)	10.8 (10.9)	0.26	10.2 (12.7)	30.4 (13.1)	<**0.01**	−1.9 (8.1)	19.6 (9.2)	<**0.01**
CESD		6.1 (7.1)	6.8 (7.4)	0.34	6.0 (6.4)	7.1 (7.7)	0.13	−0.2 (5.9)	0.3 (6.4)	0.50
PASE		159.2 (80.5)	161.9 (83.8)	0.74	160.9 (79.6)	160.6 (84.5)	0.97	1.7 (74.0)	−1.4 (65.0)	0.64
Gait speed [m/s]		1.34 (0.20)	1.33 (0.21)	0.37	1.33 (0.20)	1.26 (0.22)	<**0.01**	−0.015 (0.11)	−0.067 (0.14)	<**0.01**
Five times sit to stand [s]		10.50 (3.95)	11.53 (5.00)	**0.03**	10.42 (3.93)	12.01 (5.12)	<**0.01**	−0.034 (1.99)	0.563 (2.09)	<**0.01**
**Radiographic**										
Minimum medial JSW [mm]		3.79 (1.44)	3.70 (1.5)	0.54	3.74 (1.50)	3.57 (1.64)	0.27	−0.047 (0.43)	−0.132 (0.46)	0.06
KL grades	≥2, n (%)	490 (74.4)	101 (82.1)	0.09	509 (77.2)	105 (85.4)	0.06	29 (2.8%)	4 (3.3%)	0.82
**Physical Activities**										
Daily total activity	[$10^{4}$ count/day]	22.1 (10.6)	22.1 (11.3)	0.98	19.8 (10.5)	18.1 (9.5)	0.07	−2.2 (7.6)	−4.0 (7.6)	**0.02**
SED	[min/day]	581.3 (82.0)	572.0 (80.7)	0.24	594.9 (81.5)	584.5 (91.3)	0.24	13.7 (73.7)	12.5 (67.8)	0.87
LPA	[min/day]	277.0 (74.9)	283.9 (80.5)	0.38	254.4 (77.5)	254.8 (74.5)	0.96	−22.6 (62.6)	−29.1 (56.9)	0.25
MVPA	[min/day]	19.8 (19.4)	19.0 (20.0)	0.68	17.3 (18.3)	13.6 (16.0)	**0.02**	−2.5 (14.3)	−5.4 (14.9)	**0.049**

The values represent the mean and (SD) for the continuous variables or n (%) for the categorical variables. WOMAC—Western Ontario and McMaster Universities Osteoarthritis Index; CESD—the Center for Epidemiologic Studies Depression Scale; PASE—the Physical Activity Scale for the Elderly; SED—sedentary time; LPA—light physical activity; MVPA—moderate-to-vigorous physical activity; JSW—medial-side minimum joint space width.

## Data Availability

The data of this article are available from the authors on reasonable request.

## Supplementary Materials

The following supporting information can be downloaded at https://www.mdpi.com/article/10.3390/s26030982/s1, Figure S1: Sensor non-wearing time per 3 h intervals at the baseline; Figure S2: Longitudinal change of physical activity per 3 h intervals; Table S1: Comparison of baseline characteristics of individuals included in the study and individuals excluded due to having knee surgery; Table S2: Comparison of baseline physical activity outcomes between present study and prior studies.
