# Supplementary material for: Temporal Patterns of Wearable Accelerometer-Measured Physical Activity and Symptom Worsening in Knee Osteoarthritis: A 2-Year Longitudinal Study from the Osteoarthritis Initiative
Source: Sensors (Basel). 2026 Feb 3;26(3):982. doi: 10.3390/s26030982 (PMC12899396; doi:10.3390/s26030982)
Supplement: Supplementary file 1 [file sensors-26-00982-s001.zip › sensors-4083634-supplementary.pdf]

Supplementary Figure S1: Sensor Non-wearing time per 3-hour intervals at baseline.

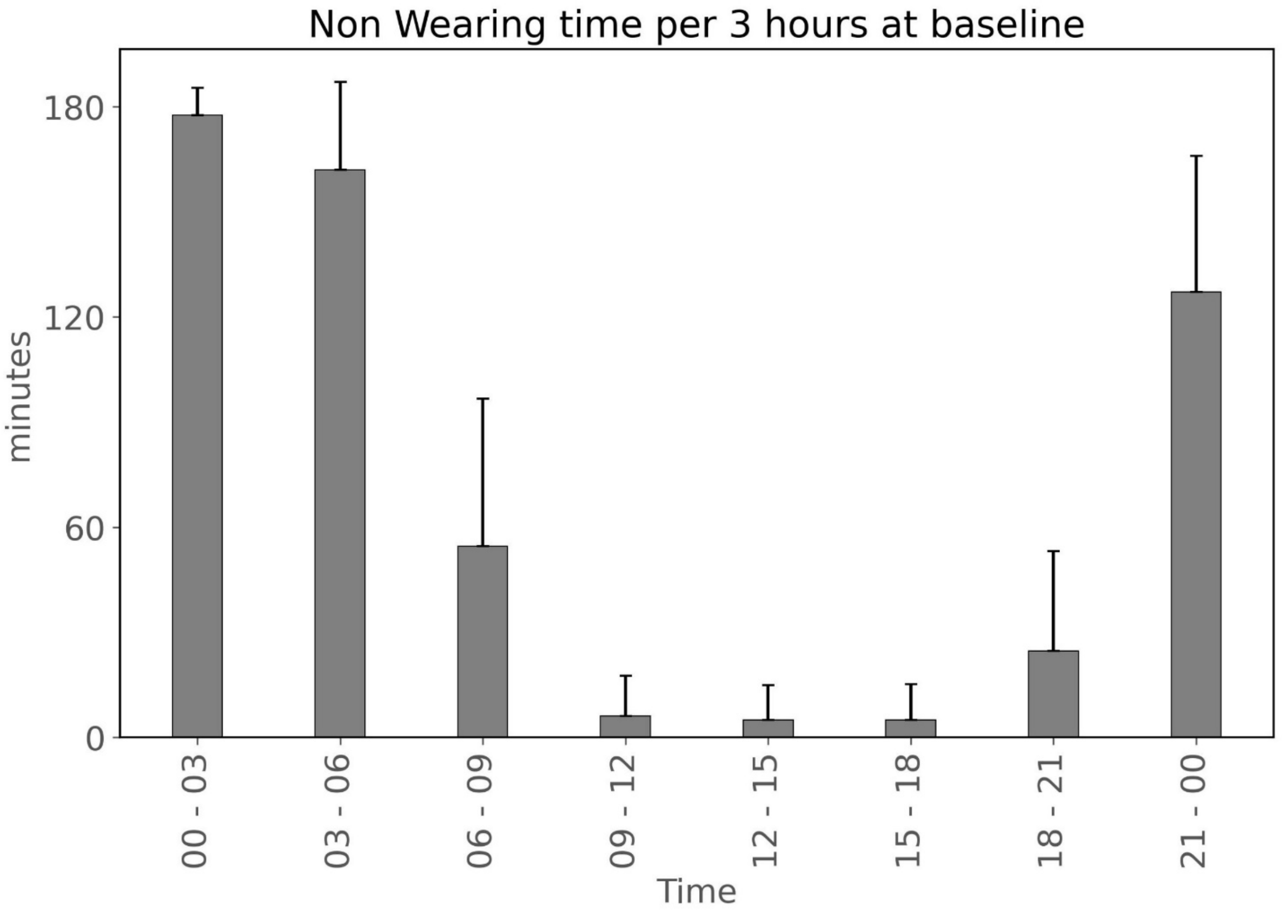

Only data from 09:00-21:00 are included in the full analysis.

Supplementary Figure S2: Longitudinal change of physical activity per 3-hour intervals

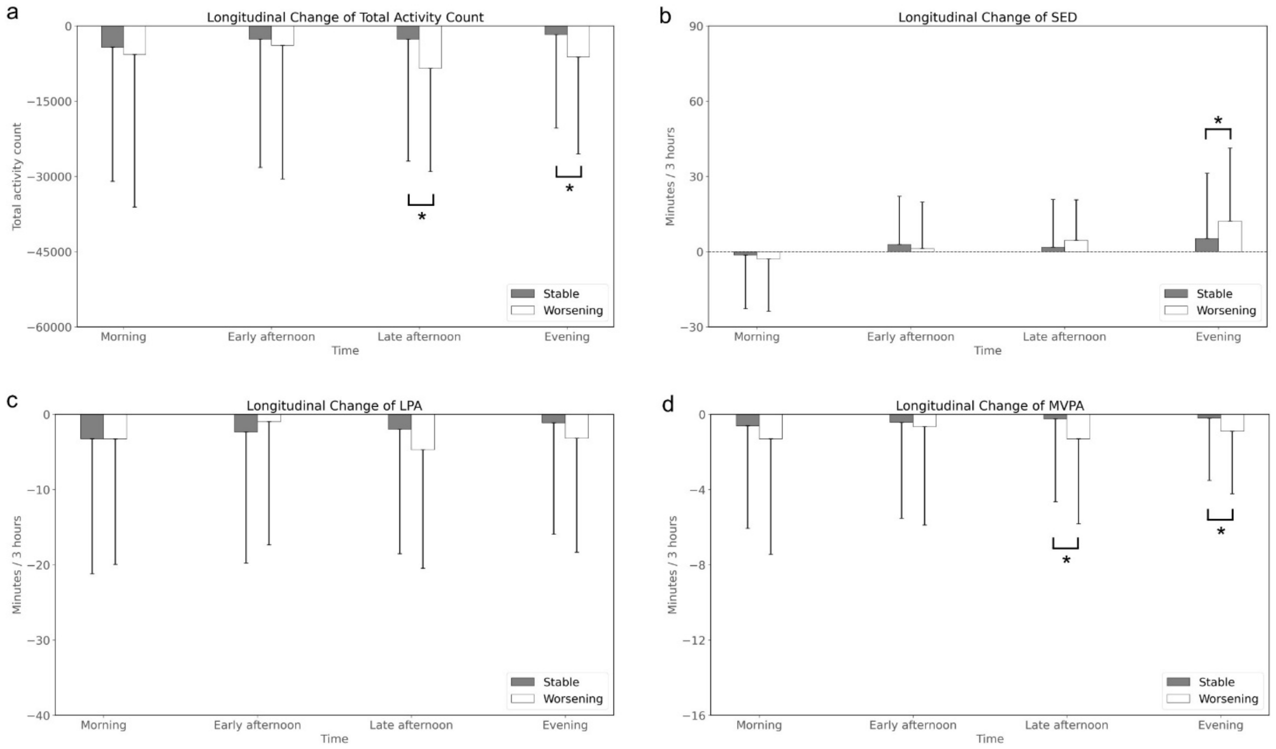

Supplementary Table S1: Comparison of baseline characteristics of individuals included in the study and individuals excluded from the study due to having knee replacement surgery.

**Demographic**		**W/O Surgery**	**W/ Surgery**	**Clinical**		**W/O Surgery**	**W/ Surgery**
Number		782	85	WOMAC total		11.9 (14.9)	24.5 (19.5)
Age		64.9 (9.1)	66.4 (8.8)	CESD		6.3 (7.2)	7.9 (6.8)
Gender	Female, n (%)	434 (55.5)	40 (47.1)	PASE		159.6 (80.9)	153.1 (81.3)
Race	White or Caucasian, n (%)	671 (85.8)	73 (85.9)	Cormorbidity ≧2	n (%)	86 (11.0)	13 (15.3)
	Black or African American, n (%)	102 (13.0)	9 (0.6)	Fall History ≧2	n (%)	117 (15.0)	11 (12.9)
	Asian, n (%)	4 (0.5)	1 (1.2)	Gait speed	[m/sec]	1.34 (0.20)	1.27 (0.19)
	Other Non-white, n (%)	5 (0.6)	2 (2.4)	Five times sit to stand	[sec]	10.66 (4.14)	13.52 (7.18)
**Physical Activity**				**Radiographic**			
Daily total activity counts	[10^4count/day]	22.1 (10.7)	19.1 (10.9)	Minimum medial JSW	[mm]	3.78 (1.45)	2.79 (1.72)
SED	[min/day]	579.8 (81.8)	591.5 (81.3)	KL grades ≧2	n (%)	591 (75.6)	52 (61.2)
LPA	[min/day]	278.1 (75.8)	260.2 (77.8)				
MVPA	[min/day]	19.7 (19.5)	14.8 (18.6)				

Individuals excluded with knee surgery have overall more pain and worse function, as well as lowered physical activity, in comparison to the included cohort. However, because the main focus of this work, we decided to include the following table in the supplementary materials

Supplementary Table S2: Comparison of baseline physical activity outcomes between present study and prior studies. In the present study, baseline activity metrics including daily total minutes in SED, LPA, and MVPA are on par with numbers reported in prior studies

	SED (min/day)	LPA (min/day)	MVPA (min/day)
Present study	579.8	278.1	19.7
Jayabalan et al. 2019	601.5	283.8	20.0
Dunlop et al. 2011, Male participants	608.2	262.2	20.7
Dunlop et al. 2011, Female participants	585.8	288.2	12.3
White et al. 2017	590.1	281.7	18.4

Jayabalan, P., Kocherginsky, M., Chang, A. H., Rouleau, G. W., Koloms, K. L., Lee, J., ... & Sharma, L. (2019). Physical activity and worsening of radiographic findings in persons with or at higher risk of knee osteoarthritis. Arthritis care & research, 71(2), 198-206.

White, D. K., Lee, J., Song, J., Chang, R. W., & Dunlop, D. (2017). Potential functional benefit from light intensity physical activity in knee osteoarthritis. American journal of preventive medicine, 53(5), 689-696.

Dunlop, D. D., Song, J., Semanik, P. A., Chang, R. W., Sharma, L., Bathon, J. M., ... & Hootman, J. M. (2011). Objective physical activity measurement in the osteoarthritis initiative: Are guidelines being met?. Arthritis & Rheumatism, 63(11), 3372-3382.
